# Melittin-MIL-2 fusion protein as a candidate for cancer immunotherapy

**DOI:** 10.1186/s12967-016-0910-0

**Published:** 2016-06-01

**Authors:** Mingjun Liu, Haitao Wang, Linjie Liu, Bin Wang, Guirong Sun

**Affiliations:** Department of Clinical Laboratory, The Affiliated Hospital of Qingdao University, 16 Jiangsu Road, Qingdao, 266003 China; Department of Public Health, Qingdao University Medical College, 38 Dengzhou Road, Qingdao, 266021 China; Key Laboratory of Medicine and Biotechnology, Department of Microbiology, Qingdao University Medical College, 308 Ningxia Road, Qingdao, 266071 China

**Keywords:** Melittin, IL-2, Mutant, Fusion protein, Cancer immunotherapy

## Abstract

**Background:**

Cytokine fusion protein that modulates the immune response holds great potential for cancer immunotherapy. IL-2 is an effective treatment against advanced cancers. However, the therapeutic efficacy of IL-2 is limited by severe systemic toxicity. Several mutants recombinant IL-2 can increase antitumor activity and minimize systemic toxicity. Melittin is an attractive anticancer candidate because of its wide-spectrum lytic properties. We previously generated a bifunctional fusion protein melittin-MIL-2, composed of melittin and a mutant IL-2. The melittin-MIL-2 inhibited the growth of human ovarian cancer SKOV3 cells in vitro and in vivo tumor growth. However, whether this antitumor effect could also be used in cancer immunotherapy was unknown. To assess its cancer immunotherapy potential, we further investigated its more effective antitumor immune response and antitumor effect against cancers of different tissue origins in vitro and in vivo.

**Methods:**

The specific IL-2 activity of the melittin-MIL-2 fusion protein was tested on the cytokine growth dependent cell line CTLL-2. The cytolytic activity was detected by standard 4-h ^51^Cr-release assays. PBMC stimulation in response to the melittin-MIL-2 was determined by IFN-γ release assay. We observed the cancer cell proliferation of different tissue origins by MTT assay. The ability of melittin-MIL-2 to inhibit tumor growth in vivo was evaluated by using human liver (SMMC-7721 cancer cells), lung (A549 cancer cells) and ovarian (SKOV3 cancer cells) cancer xenograft models. To assess the immunity within the tumor microenvironment, the level of some cytokines including IFN-γ, TNF-α, IL-12 and IL-4 was analyzed by ELISA. We injected the MDA-MB-231 cells and the melittin-MIL-2 into mice, and the anti-metastatic effect was examined by counting nodules in the lung.

**Results:**

The melittin-MIL-2 was more effective in inducing T cell and NK-cell cytotoxicity. The fusion protein significantly increased IFN-γ production in PBMCs. In vitro, the melittin-MIL-2 mediated immune cells killing or directly killed the cancer cell lines of different tissue origins. In vivo, the fusion protein exhibited stronger inhibition on the growth of transplanted human tumors compared to rIL-2. The melittin-MIL-2 treatment promoted the IFN-γ secretion in tumor tissues and decreased the immunosuppressive cells in vivo. Furthermore, the fusion protein reduced lung metastasis of breast cancer.

**Conclusions:**

This study provides the evidence that the melittin-MIL-2 can produce stronger immune stimulation and antitumor effects, and the fusion protein is a potent candidate for cancer immunotherapy.

## Background

Immunotherapy is one of the most promising approaches for future cancer therapy [1–3]. Cancer immunotherapy is to augment a more effective antitumor immune response through increasing active immune cells and reversing tumor-induced immunosuppression. Various immunotherapy methods, including cytokines, tumor vaccines and monoclonal antibodies have exhibited potential therapeutic potency in both animal models and cancer patients. The satisfactory results of cytokine-based immunotherapy are being observed [1–3].

Cytokines have great potential in cancer immunotherapy [2]. Interleukin-2 (IL-2) is one of the most successful cytokines applied in tumor immunotherapy, which plays a key role in immune regulation and T cell proliferation [4]. IL-2 is an effective option against advanced cancers. However, high dosage IL-2 can lead to obvious systemic side effects, such as fever, renal dysfunction, capillary leakage and hypotension [5, 6]. Furthermore, attempts to minimize toxicity by lowering the IL-2 dosage have resulted in declined antitumor efficacy as well. To improve the therapeutic efficacy of IL-2, it is critical to develop novel methods without leading to severe side effects. Several mutants recombinant IL-2 (MIL-2) can augment the antitumor effect and reduce systemic toxicity [7–9]. We previously constructed a functional mutant IL-2 using site-directed mutagenesis [10]. Current research is being aimed at developing combination strategies having enhanced antitumor effects with less related toxicities [11].

Melittin, the major component of European bee venom, has immunoregulatory activity and anticancer effect [12–18]. Bee venom can enhance T lymphocytes esterase expression in S180 sarcoma mouse and increase T lymphocyte function [12]. Melittin augments Th1 cells function and is used for the therapy of low immune function, cancer and viral infection [13]. Melittin inhibits proliferation of different tissue origins cancer cells [14–19]. Thus, melittin is a potent antitumor candidate.

In a previous study, we have successfully generated a bifunctional fusion protein melittin-MIL-2, composed of melittin and a mutant IL-2. The melittin-MIL-2 inhibited the growth of human ovarian cancer SKOV3 cells in vitro and in vivo tumor growth [20]. However, whether this antitumor effect could also be used in cancer immunotherapy was unknown. In this study, we further investigated its more effective antitumor immune response and antitumor effects against cancers of different tissue origins, and evaluated its cancer immunotherapy potential.

## Methods

### Reagents, cell lines and animals

Recombinant melittin-MIL-2 fusion protein was generated and stored in our laboratory. Recombinant human IL-2 (rhIL-2) was purchased from National Institute for the Control of Pharmaceutical and Biological Products (Beijing, China). Melittin was purchased from Nanning Innovation and Technology Pharmaceutical Company (Guangxi, China). Interleukin-2-dependent cytotoxic T lymphocyte (CTLL-2) cell line was cultured in RPMI 1640 supplemented with 10 % fetal bovine serum (FBS) and IL-2. Cytokines enzyme linked immunosorbent assay (ELISA) kits were purchased from Shanghai Westang Biotechnology Company Limited. (Shanghai, China). These cytokines include interferon gamma (IFN-γ), tumor necrosis factor alpha (TNF-α), interleukin-12 (IL-12) and interleukin-4 (IL-4). Human liver cancer cells SMMC-7721, lung cancer cells A549, ovarian cancer cells SKOV3 and breast cancer cells MDA-MB-231 were obtained from Cell Bank of Chinese Academy of Science, Shanghai, China. BALB/c mice (6 week-old) were purchased from Institute of Laboratory Animal Sciences, Chinese Academy of Medical Sciences, Beijing, China.

### Proliferation assay and cytotoxicity assay

The specific IL-2 activity of the melittin-MIL-2 fusion protein was assessed on the cytokine growth dependent cell line CTLL-2. Various concentrations (8.0, 2.0, 0.5, 0.125 µM) of the melittin-MIL-2 were incubated for 48 h with 2 × 10^4^ CTLL-2 cells that had been starved of IL-2. One μ Ci of [^3^H] thymidine was added to the medium for the last 18 h and cell proliferation was determined by [^3^H] thymidine incorporation. The same dilutions of rhIL-2 were used as standard. Phosphate buffer saline (PBS) was used as a negative control. We prepared peripheral blood mononuclear cells (PBMCs), CD4^+^, CD8^+^ T cells and NK cells as described in previous study [21]. PBMCs were obtained from three normal, healthy donors (Donor A, B, C). The experiments were approved by the Institutional Review Board of The Affiliated Hospital of Qingdao University. Mononuclear cells were isolated by Ficoll-Hypaque (Pharmacia Biotech, Uppsala, Sweden) gradient separation and cultured in six-well plates (5 × 10^6^ cells/5 ml/well) in a humidified incubator with 5 % CO_2_ at 37 °C. Culture was maintained in AIM-V medium (Life Technologies, Rockville, USA). At the same time, the medium was added same concentration (2.0 µM) melittin-MIL-2 or rhIL-2 or melittin. PBS was used as a negative control. CD4^+^, CD8^+^ T cells and NK cells were enriched from PBMCs by negative immunomagnetic selection. PBMCs were labeled with the enrichment antibody cocktails for human T cells (CD4^+^, CD8^+^) and NK cells, and then with magnetic colloid according to the product insert supplied by StemCell Technologies (Vancouver, BC, Canada). The cell suspension was then passed through a high-gradient magnetic column (0.5-inch diameter) of stainless steel mesh to remove unwanted magnetically labeled cells. Cell purity was determined to be 85 ± 5 % by flow cytometry. Enriched cells were cultured in 24-well plates (1 × 10^6^ cells/ml/well) in AIM-V medium. At the same time, the medium was added same concentration (2.0 µM) melittin-MIL-2 or rhIL-2 or melittin. PBMCs, CD4^+^, CD8^+^ T cells and NK cells were cultured for 3–5 days and used as effector cells. Cells from hepatocellular carcinoma cell line SMMC-7721 were used as target tumor cells. The cytolytic activity was detected by standard 4-h ^51^Cr-release assays. We washed the targets free of excess ^51^Cr and plated the targets in 96-well plate at 5 × 10^3^ cells/well. Then, we added the effector cells to the wells at various effector-to-target ratios. The radioactivity released from the lysed tumor cells was measured by a gamma counter. The percentage of cytolysis was converted to lytic units (LU_20_/10^7^cells). Each of these experiments was repeated three times.

### IFN-γ enzyme linked immunosorbent assay (ELISA)

Peripheral blood mononuclear cells stimulation in response to the melittin-MIL-2 fusion protein was determined by IFN-γ release assay. The ELISA was conducted with procedures described in the reagent instruction manual supplied by Shanghai Westang Biotechnology Company Limited. Here, we performed the experimental condition with the melittin-MIL-2 fusion protein as described for the PBMC proliferation assays. PBMCs were obtained from three normal, healthy donors (Donor A, B, C). Then, supernatant was extracted 5 days after PBMC addition and concentration of IFN-γ was analyzed by ELISA. These experiments were repeated three times.

### Cancer cells proliferation assay

To observe the dose-dependent inhibition of cancer cell proliferation, SMMC-7721 hepatocellular carcinoma cells (2 × 10^4^) were incubated with increasing concentrations of melittin-MIL-2 (50, 100, 150, 200 μg/ml). PBS was used as a negative control. Cell proliferation was determined in hexaplicates after 3 days, using the MTT assay. To evaluate the growth inhibition of cancer cell lines from different tissue origins, liver cancer cells (SMMC-7721), mammary cancer cells (MDA-MB-231), ovary cancer cells (SKOV3), gastric cancer cells (SGC-7901) and lung cancer cells (A549) were used. We plated cancer cells in 96-well plates (2000 cells/well) and added the melittin-MIL-2 (100 μg/ml) at the beginning of the incubation. PBS was used as negative control in all cancer cell proliferation experiments. Cell proliferation was determined by the MTT assay. Recombinant IL-2 and melittin were used as experimental controls in ovarian cancer cells SKOV3 proliferation experiment. We determined cell proliferation after 24, 48 or 72 h using the MTT assay. During the incubation, we checked the dishes every day and replenished media. These assays were repeated three times.

### Tumor challenge and treatment experiments

In the experiments, human liver (SMMC-7721 cancer cells), lung (A549 cancer cells) and ovary (SKOV3 cancer cells) cancer xenograft models were used. These cancer cells (SMMC-7721: 5 × 10^6^ cells per animal, A549: 5 × 10^6^ cells per animal, SKOV3: 2 × 10^5^ cells per animal) were subcutaneously injected into female BALB/c mice (6 week-old). When tumors became palpable (5–7 days), the mice were randomized into groups (5 mice per group) and administered intraperitoneally at the indicated time points with the melittin-MIL-2 (200 μmol per animal) or rIL-2 (200 μmol per animal) or melittin (200 μmol per animal). Tumor volumes were measured twice a week. As soon as mice produced ascites and had a weight increase >30 %, they were sacrificed. The xenograft model (liver SMMC-7721 cancer) was used to observe the mice survival. We carefully observed and recorded the survival of each mouse, and calculated overall survival.

### Cytokine assay in the tumor tissue

In order to evaluate the immunity efficacy of melittin-MIL-2 on the breast tumor tissue, we killed the mice 30 days after tumor challenge and gathered the tumor tissue of each mouse. Then, we homogenized the tumor tissue in PBS and tested cytokine concentration of each sample. The equal amount of each sample was detected for IFN-γ, TNF-α, IL-12 and IL-4 using ELISA kits obtained from Shanghai Westang Biotechnology Company Limited (Shanghai, China) according to the manufacturer’s instructions. We computed the concentration of each cytokine relative to respective standard curves. Each group includes six mice.

### Determination of anti-metastasis activities

This experiment was conducted as described previously [22, 23]. MDA-MB-231 cells were resuspended in culture media at a density of 1.5 × 10^6^ per 150 μL and implanted into the mammary fat pad of the female BALB/c mice (6 week-old). When a distinct tumor mass (4–5 mm in diameter) was detectable (for 3 weeks), the mice were equally randomized into two groups (10 mice per group): the melittin-MIL-2 (200 μmol per mouse) group and control group. The control group received 0.9 % normal saline. The melittin-MIL-2 was then administered (once every other day) for 60 days by intravenous injection. Tumor size was measured once every week. Twenty-four hours after the final administration, animals were weighed and sacrificed. Tumors were weighed and collected. Then, the lungs were removed, washed and fixed with Bouin’s solution for 24 h to facilitate the counting of tumor nodules as described previously [23]. The number of tumor nodules on the whole surface of the lungs was counted using a microscope.

### Statistical analyses

Data were indicated as mean ± SD. Differences between groups were analyzed for statistical significance using the Student’s t test. A *p* value less than 0.05 represented a statistically significant difference. SPSS Version 19.0 for Windows software (SSPS Inc., Chicago, USA) was used for the calculation.

## Results

### The melittin-MIL-2 induced proliferation and stronger cytolytic activity of activated lymphocytes

**Fig. 1:**
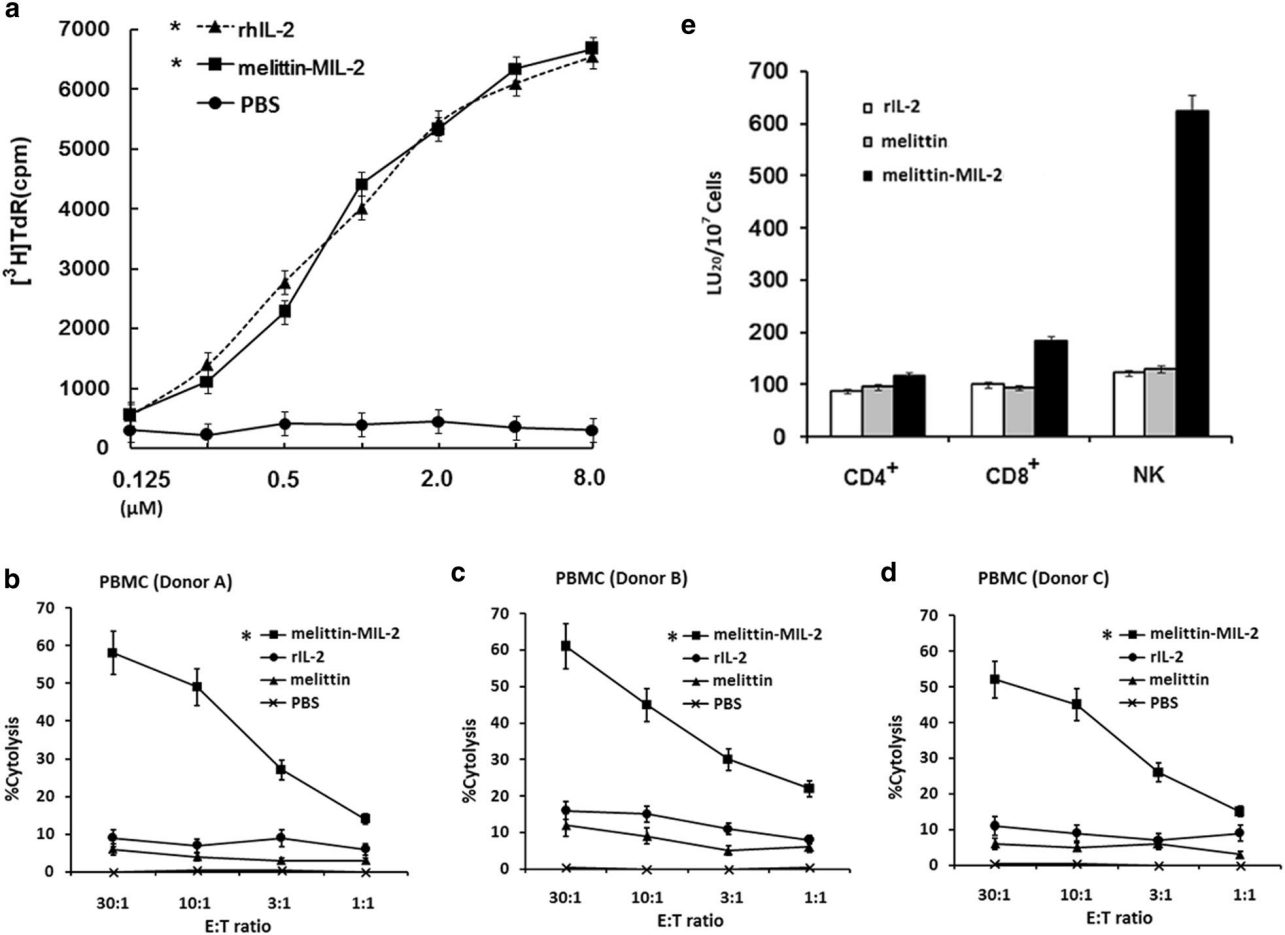
The melittin-MIL-2 induced proliferation and stronger cytolytic activity of activated lymphocytes. **a** The IL-2 activity of melittin-MIL-2 fusion protein was tested by its ability to stimulate proliferation of CTLL-2 cells. Various concentrations (8.0, 2.0, 0.5, 0.125 µM) of the fusion protein were incubated for 48 h with 2 × 10^4^ CTLL-2 cells that had been starved of IL-2. One μ Ci of [^3^H] thymidine was added to the medium for the last 18 h, and cell proliferation was determined by [^3^H] thymidine incorporation. The same dilutions of rhIL-2 were used as standard. PBS was used as negative control (*p < 0.05). **b**–**e** The melittin-MIL-2 induced stronger cytolytic activity of PBMCs, specifically NK cells. **b**–**d** Representative standard 4-h ^51^Cr-release data were shown against the hepatocellular carcinoma cell line SMMC-7721. When the melittin-MIL-2 (2.0 µM) was used, the cytolytic activity was significantly greater compared with rIL-2 and melittin alone (*p < 0.01) at a 30:1 effector-to-target ratio (E:T). **e** Enriched T cells (CD4^+^, CD8^+^) and NK cells were cultured for 3 days and tested for cytolytic activity. Increase in cytolytic activity was most notable in NK cells. When the fusion protein (2.0 µM) was used, cytolytic activity of NK cells increased sixfold compared with those cultured with rIL-2 or melittin alone

To evaluate the IL-2 activity of the melittin-MIL-2, we compared the fusion protein with rIL-2 for its ability to induce proliferation of CTLL-2 (Fig. 1a). PBMCs were cultured for 5 days at various concentrations of the melittin-MIL-2, rIL-2 and melittin and their cytolytic activities were analyzed against hepatocellular carcinoma cell line SMMC-7721. The fusion protein significantly enhanced the cytolytic activity of PBMCs compared with the same levels of rIL-2 or melittin (Fig. 1b–d). When the melittin-MIL-2 was used, the cytolytic activity was significantly greater compared with rIL-2 or melittin (*p < 0.01) at a 30:1 effector-to-target ratio (Fig. 1b–d). When tested on respective T cells (CD4^+^, CD8^+^) and NK cells, a significant increase in cytolytic activity was most conspicuous in the NK cell population. When the melittin-MIL-2 was used, the cytolytic activity of NK cells augmented sixfold compared with those cultured with rIL-2 or melittin (Fig. 1e). Here, melittin-MIL-2 showed similar activity than rIL-2 and stronger cytolytic activity than rIL-2.

### The melittin-MIL-2 promoted the production of IFN-γ

**Fig. 2:**
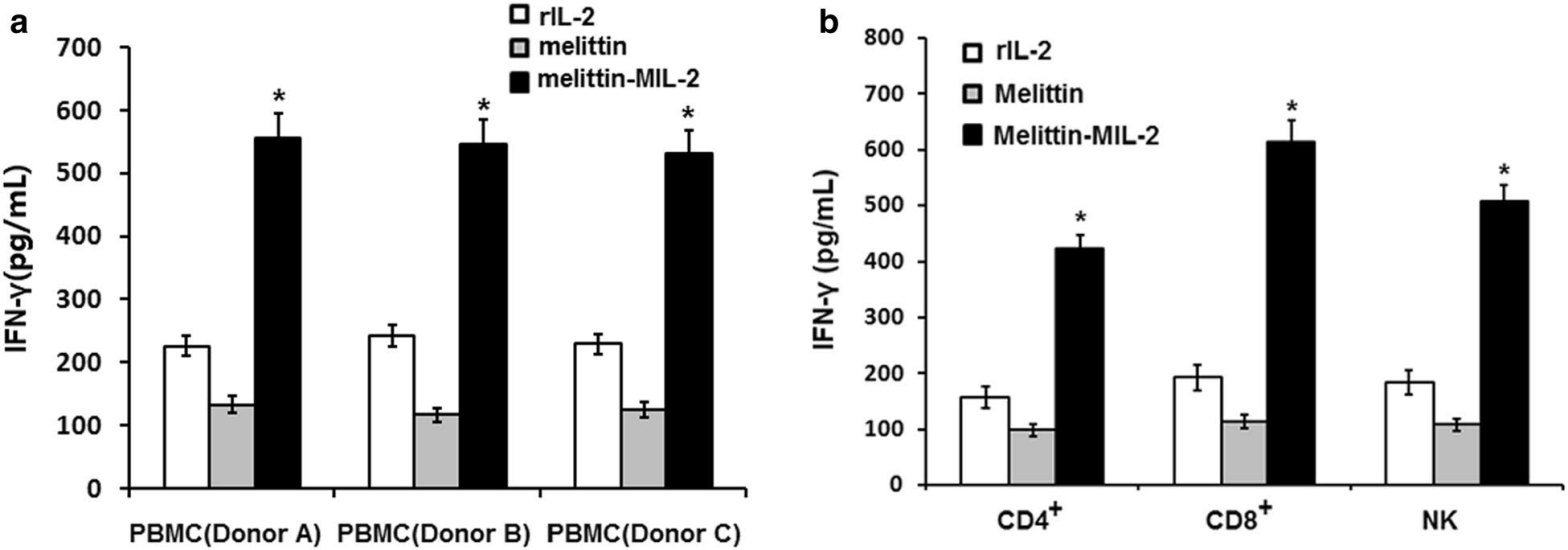
The melittin-MIL-2 promoted the production of IFN-γ. **a** Representative IFN-γ ELISA for PBMCs cultured for 3 days with the melittin-MIL-2 (2 µM) or rIL-2 (2 µM) and melittin (2 µM) alone. PBMCs were obtained from 3 normal, healthy donors (Donor A, B, C). **b** When tested on isolated T cells (CD4^+^, CD8^+^) and NK cells, increase in IFN-γ production was observed in all cells treated with the melittin-MIL-2. *p < 0.01 compared to control (rIL-2 or melittin)

We compared the level of IFN-γ in culture supernatants of PBMCs that were exposed to various concentrations of the melittin-MIL-2 fusion protein. One representative IFN-γ ELISA was shown in Fig. 2. Our findings indicated a significant increase in the production of IFN-γ by the PBMCs in melittin-MIL-2 group compared to rIL-2 or melittin (Fig. 2a). When the melittin-MIL-2 fusion protein was tested on isolated T cells (CD4^+^, CD8^+^) and NK cells, an increase in the production of IFN-γ was observed in all of these cells (Fig. 2b).

### The melittin-MIL-2 inhibited proliferation of cancer cell lines of different tissue origins

**Fig. 3:**
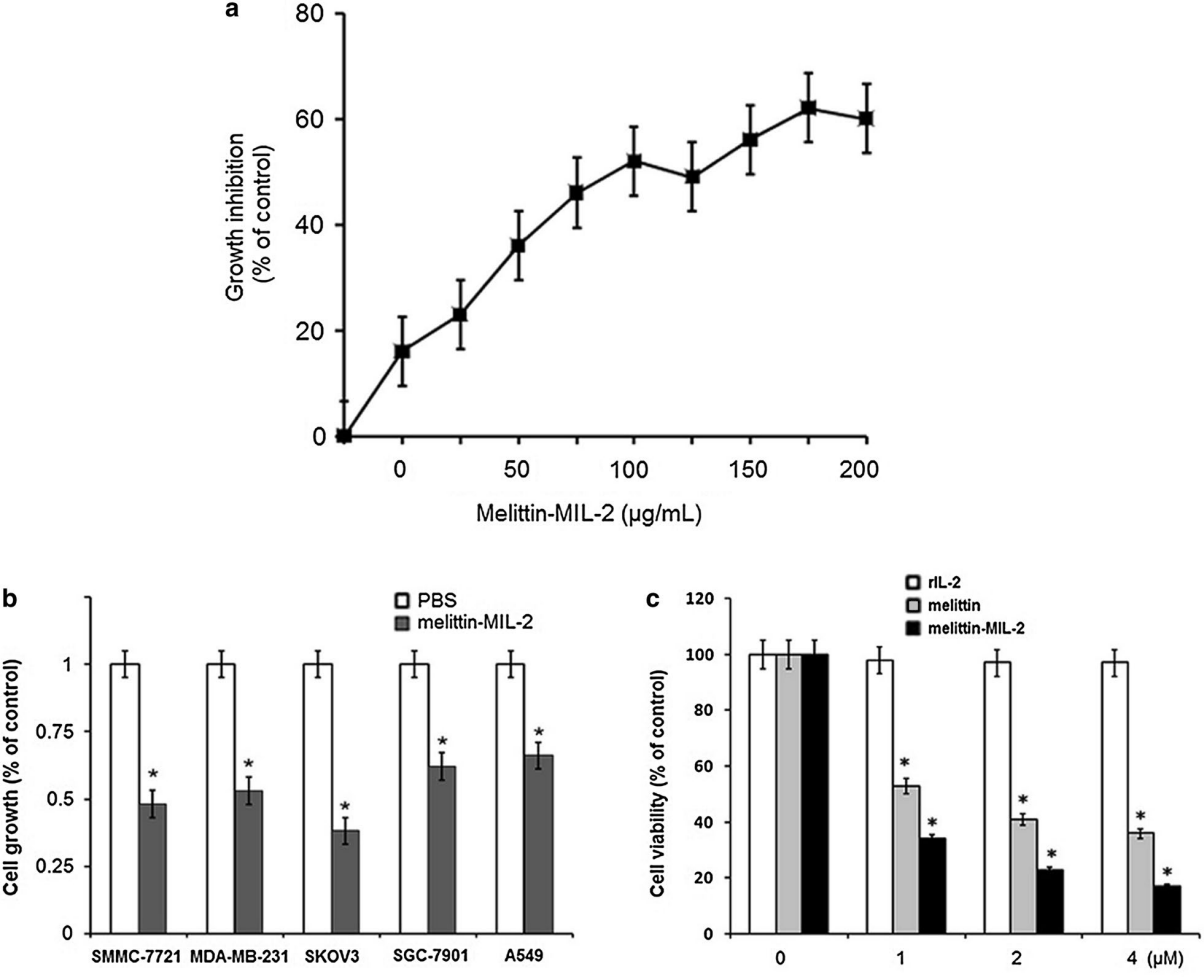
The melittin-MIL-2 inhibited proliferation of human tumor cells. PBS was used as negative control in all cancer cell proliferation experiments. Cell proliferation was determined by the MTT assay. **a** To observe the dose-dependent inhibition of cancer cell proliferation, SMMC-7721 hepatocellular carcinoma cells (2 × 10^4^) were incubated with increasing concentrations of melittin-MIL-2. Cell proliferation was determined in hexaplicates after 3 days. **b** To assess the growth inhibition of cancer cell lines from different tissue origins, liver cancer cells (SMMC-7721), mammary cancer cells (MDA-MB-231), ovary cancer cells (SKOV3), gastric cancer cells (SGC-7901) and lung cancer cells (A549) were used. We plated cancer cells in 96-well plates (2000 cells/well) and added the melittin-MIL-2 (100 μg/ml) at the beginning of the incubation. The indicated cancer cells were incubated for 3 days with melittin-MIL-2 and cell proliferation was determined (*p < 0.05). **c** The melittin-MIL-2 inhibited cell proliferation of ovarian cancer cells SKOV3 in a concentration-dependent manner; rIL-2 did not inhibit cell proliferation of SKOV3. The most effective inhibitory melittin-MIL-2 concentration was 4 μM, causing growth inhibition of 74 % (*p < 0.05)

We observed the dose-dependent inhibition of proliferation, up to 60 %, when SMMC-7721 hepatocellular carcinoma cells were cultured with increasing levels of melittin-MIL-2 (Fig. 3a). Moreover, we found the similar inhibitory effects in four additional cancer cell lines of mammary, lung, ovary and gastric origins (Fig. 3b). Our findings demonstrated that melittin-MIL-2 and melittin inhibited cell proliferation of ovarian cancer cells SKOV3 in a concentration-dependent manner; rIL-2 did not inhibit SKOV3 cell proliferation (Fig. 3c). The growth curves of SKOV3 cells were analyzed, following incremental increases in level of melittin-MIL-2 for 24, 48 and 72 h. In all tested levels, treatment for 48 h was most effective. The number of SKOV3 cells was represented as a percentage of those cells of the control (0 μM melittin-MIL-2) cases. The most effective inhibitory concentration of the melittin-MIL-2 was 4 μM, leading to growth inhibition of 74 %.

### The melittin-MIL-2 inhibited tumor growth and exhibited enhanced antitumor activity compared to rIL-2 in vivo

**Fig. 4:**
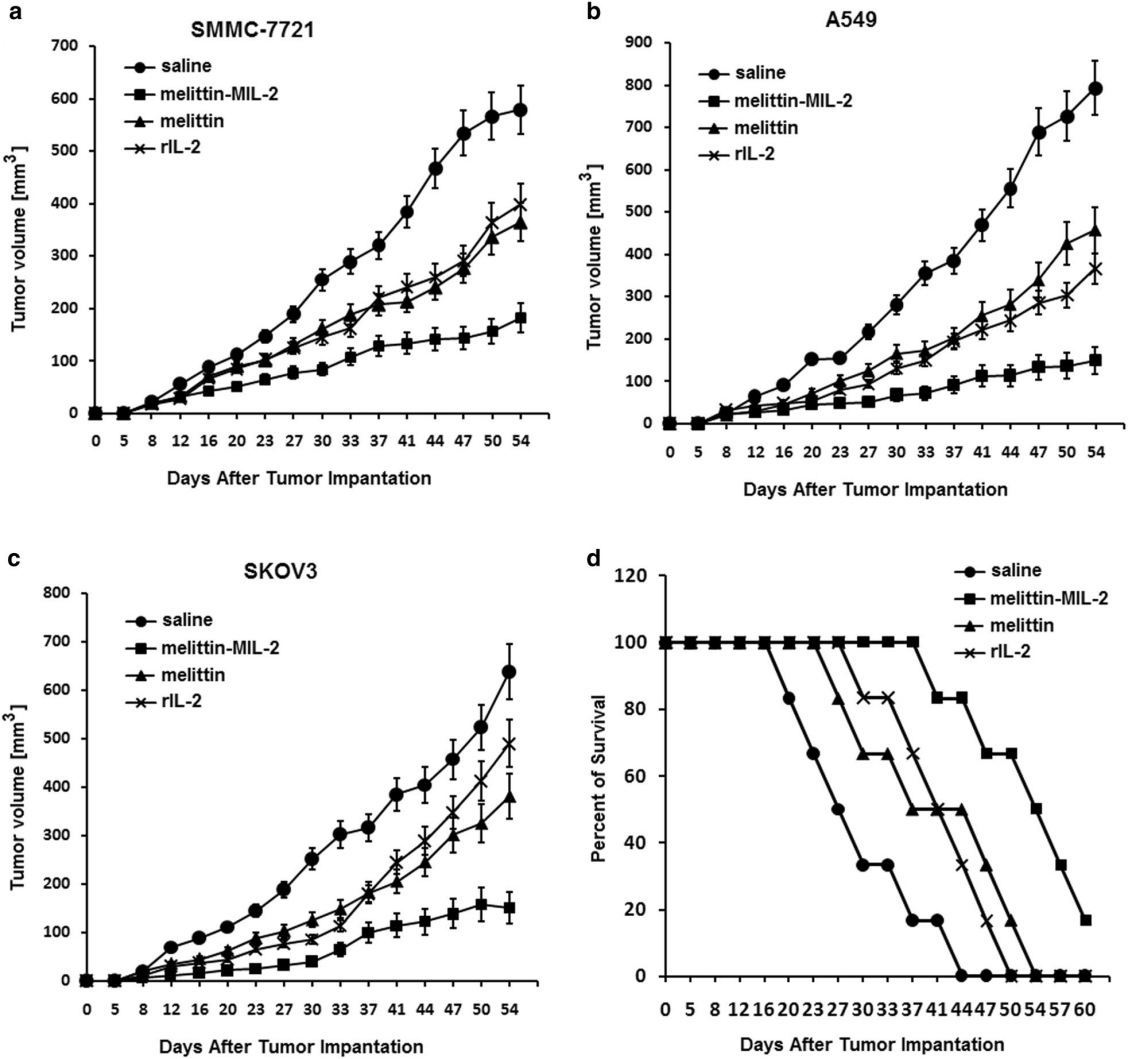
The melittin-MIL-2 inhibited tumor growth and exhibited enhanced antitumor activity compared to rIL-2 in vivo. These cancer cells (SMMC-7721: 5 × 10^6^ cells per animal, A549: 5 × 10^6^ cells per animal, SKOV3: 2 × 10^5^ cells per animal) were subcutaneously injected into female BALB/c mice (6 week-old). When tumors became palpable (5–7 days), the mice (n = 5 per group) were randomized into groups and administered intraperitoneally at the indicated time points with the melittin-MIL-2 (200 μmol per animal) or rIL-2 (200 μmol per animal) or melittin (200 μmol per animal). Tumor volumes were measured twice a week. As soon as mice produced ascites and had a weight increase >30 %, they were killed. The xenograft model (liver SMMC-7721 cancer) was used to observe the mice survival. We carefully observed and recorded the survival of each mouse, and calculated overall survival. **a** After day 16, mean tumor volumes among treatment groups were statistically different (*p* < 0.05). Melittin-MIL-2 therapy was conspicuously different than control (saline) from day 16 to the end of the experiment (*p* < 0.05). Moreover, melittin-MIL-2 treatment was significantly different than rIL-2 treatment from day 20 to the conclusion of the experiment (*p* < 0.05). **b**, **c** After day 12, mean tumor volumes among treatment groups were statistically different (*p* < 0.05). Melittin-MIL-2 therapy was significantly different than control from day 12 to the conclusion of the experiment (*p* < 0.05). Furthermore, melittin-MIL-2 therapy was evidently different than rIL-2 therapy from day 23 to the end of the experiment (*p* < 0.05). **d** The survival time among the mice treated with melittin-MIL-2 was higher than other groups (*p* < 0.05)

The ability of melittin-MIL-2 to inhibit growth of cultured cancer cells indicated an antitumor activity in animals. This was evaluated by using human liver (SMMC-7721 cancer cells), lung (A549 cancer cells) and ovary (SKOV3 cancer cells) cancer xenograft models. The melittin-MIL-2 treatment was started only after subcutaneous lumps became palpable. The melittin-MIL-2 induced partial inhibition of all the three xenograft models (Fig. 4). We observed no significant weight loss in these experiments. Saline injections were used in a control group. There were no differences in tumor volumes among the different groups up to day 16 (Fig. 4a). After day 16, mean tumor volumes among treatment groups were statistically different (*p* < 0.05) (Fig. 4a). The melittin-MIL-2 treatment was conspicuously different than control from day 16 to the end of the experiment (*p* < 0.05) (Fig. 4a). Moreover, the melittin-MIL-2 treatment was significantly different than rIL-2 treatment from day 20 to the conclusion of the experiment (*p* < 0.05) (Fig. 4a). We observed no differences in tumor volumes among the different groups up to day 12 (Fig. 4b, c). After day 12, mean tumor volumes among treatment groups were statistically different (*p* < 0.05). The melittin-MIL-2 therapy was significantly different than control from day 12 to the conclusion of the experiment (*p* < 0.05). Furthermore, the melittin-MIL-2 therapy was evidently different than rIL-2 therapy from day 23 to the end of the experiment (*p* < 0.05). To analyze the effect of melittin-MIL-2 treatment for the duration of survival time after tumor challenge, we examined the number of living mice daily for 60 days. Each group contained six mice. Survival evaluation suggested increase in the survival rate of melittin-MIL-2 treated mice compared to other treated groups. The survival time among the mice treated with melittin-MIL-2 was higher than other groups (*p* < 0.05) (Fig. 4d). In short, the melittin-MIL-2 treatment contributed to an antitumor activity towards several different xenograft models and demonstrated enhanced antitumor activity compared to rIL-2.

### The melittin-MIL-2 treatment shaped a local immunostimulatory microenvironment

**Fig. 5:**
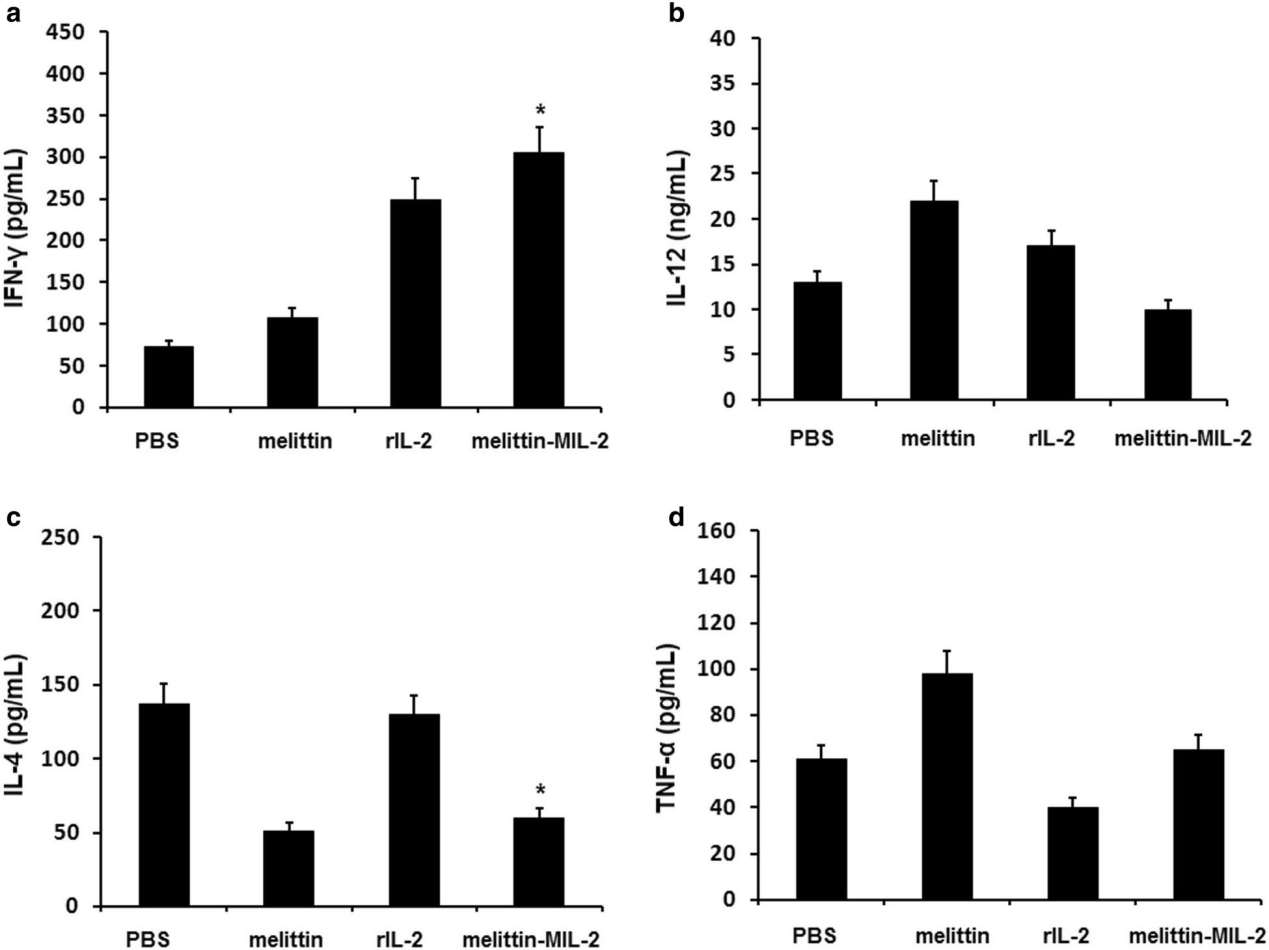
The melittin-MIL-2 treatment shaped a local immunostimulatory microenvironment. **a** The level of IFN-γ significantly increased in the treated mice with melittin-MIL-2 compared to PBS group (*p* < 0.05). **c** The level of IL-4 significantly reduced in the treated mice with melittin-MIL-2 compared to PBS group (*p* < 0.05). **b**, **d** No significant change was observed in quantity of IL-12 and TNF-α among mice treated melittin-MIL-2

Immunity within the tumor microenvironment acts as a key factor for the eradication of cancer. To assess the immunity efficacy, we analyzed the concentrations of some cytokines including IFN-γ, TNF-α, IL-12 and IL-4 by ELISA. The findings from cytokine assays were observed (Fig. 5). The level of IFN-γ among mice treated with melittin-MIL-2 was significantly higher than PBS group (*p* < 0.01) and melittin group (*p* < 0.05) (Fig. 5a). The level of IFN-γ among mice treated with rIL-2 was also higher than PBS group (*p* < 0.01) and melittin group (*p* < 0.05) (Fig. 5a). Furthermore, the level of IFN-γ in the treated mice with melittin was not significantly different from those treated with PBS (Fig. 5a). Data on IL-12 evaluation indicated no significant difference between PBS-treated group and other treated groups except in the melittin-treated group. Moreover, the IL-12 secretion in melittin-MIL-2 group was lower than rIL-2 and melittin groups (*p* < 0.05) (Fig. 5b). In spite of a significant decline in the level of IL-4 in the mice treated with melittin-MIL-2 (compared with the PBS- and rIL-2-treated groups, *p* < 0.01), we found no difference between melittin-MIL-2 and melittin groups or between PBS and rIL-2 groups in terms of IL-4 level (Fig. 5c). Additionally, there was no significant difference in the TNF-α level after treatment with melittin-MIL-2 and PBS. The TNF-α concentration in the melittin-MIL-2 group was lower than melittin group (*p* < 0.01) but higher than rIL-2 (*p* < 0.01) (Fig. 5d).

### The melittin-MIL-2 exhibited anti-metastasis activities in an animal model

**Fig. 6:**
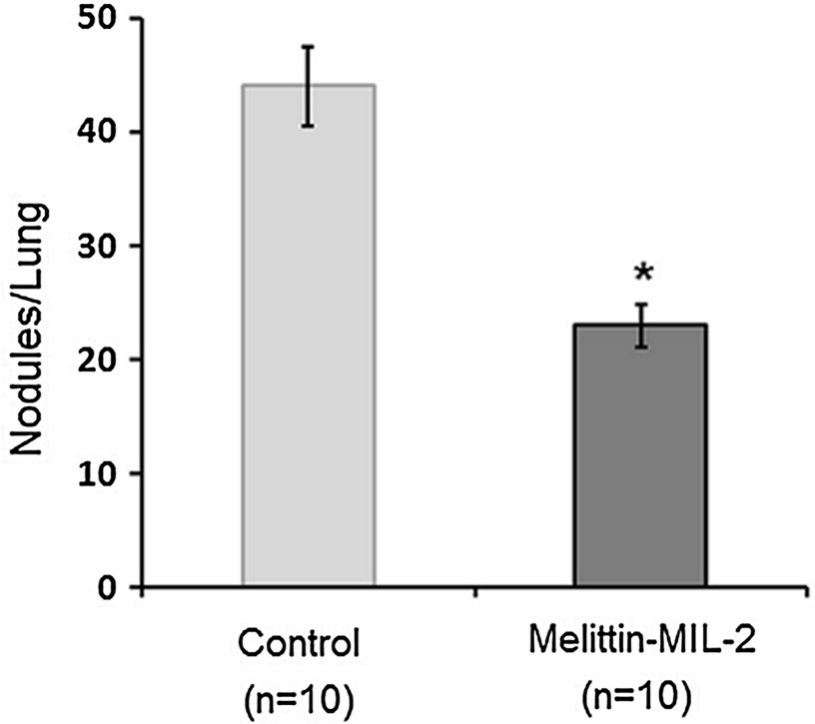
The melittin-MIL-2 reduced lung metastasis of MDA-MB-231 mammary cancer cells. The number of tumor nodules on the whole surface of the lungs was counted using a microscope. The administered melittin-MIL-2 reduced metastasis to the lung by 48 % (*p < 0.05)

To assess the effect of the melittin-MIL-2 on metastasis, we used MDA-MB-231 cells, which were initially analyzed in vitro using a cell proliferation assay. When incubated with the melittin-MIL-2, the cells showed a growth-inhibitory effect (Fig. 3b). This anti-metastasis efficacy was tested by using the melittin-MIL-2 and the MDA-MB-231 cells. We examined metastasis by counting nodules in the lung. The administered melittin-MIL-2 reduced metastasis to the lung by 48 % (Fig. 6).

## Discussion

Some studies have shown that it is possible to generate a biologically active bifunctional molecule comprising of a toxin and a cytokine [24–26]. The antitumor effects of bifunctional cytokine fusion proteins are mainly dependent on the molecular constitution, cytokine combination, and tumor animal model. In a previous study, we have successfully generated a bifunctional fusion protein melittin-MIL-2 that combines melittin and a mutant IL-2 into a single molecule [20]. Each component retained its functionality, thus simultaneous stimulation of the mutant IL-2 and the melittin on target cells, accompanied by their respective function, was obtained. Therefore, this approach of cancer immunotherapy is feasible and promising.

Research suggests that several mutants recombinant human IL-2 (MIL-2) can enhance antitumor activity and reduce toxicity [8–11]. Thus, we previously constructed a functional mutant IL-2 (Arg88/Ala125) using site-directed mutagenesis [10]. In an attempt to reduce the side effects of IL-2 immunotherapy, the current study is focusing on developing combination treatments with additive antitumor effects and decreased toxicity [11]. In this study, the IL-2 activity of melittin-MIL-2 was compared with rIL-2 for its ability to induce CTLL-2 proliferation. The results showed that the fusion protein retained its IL-2 functional activity. In the present study, the in vitro effects of the melittin-MIL-2 on human lymphocyte populations were explored. We observed significant increases in proliferation, cytotoxicity and IFN-γ production in PBMCs. When analyzed on isolated T cells (CD4^+^, CD8^+^) and NK cells, a notable increase in cytolytic effect was most significant in the NK cells. Substantial up-regulation of IL-2 receptor ɑ in NK cells may partly explain this mechanism. Several cytokines, such as IL-2, IL-12 and IL-18, are potent inducers of IFN-γ in NK and T cells [27–31]. The tumoricidal effects in vivo of IFN-γ result either from a direct action on tumor cells or indirectly via the activation of several effector mechanisms. These include stimulation of T- and NK-cell activity, stimulation of MHC antigen expression, and macrophage activation [28]. In this study, an increase in IFN-γ production in PBMCs resulted from use of the melittin-MIL-2 fusion protein. These findings showed that whether injected systemically or locally at the site of tumor, the activation, expansion and possibly the survival of effector cells that interact with tumor targets could result from the enhanced effect of the melittin-MIL-2, thus increasing the antitumor response. Indeed, we should directly compare the activity of the melittin-MIL-2 with the activity of the mutated form of IL-2 in the present experiments. However, we found that the MIL-2 had identical functional property to the wild type hIL-2 in our previous study [10]. Accordingly, we would like to incorporate into our future study to directly compare the activity of the fusion construct melittin-MIL-2 with the activity of the mutated form of IL-2.

Melittin is a small linear peptide consisting of 26 amino acids [32]. It has been reported that melittin has multiple effects, such as antivirus, antibacteria and anti-inflammation, in various cell types [32–34]. Moreover, previous studies have demonstrated that melittin can induce cell cycle arrest, growth inhibition, and apoptosis in various cancer cells [15, 33–35]. Melittin is a desirable anticancer candidate because of its broad-spectrum lytic properties. Here, proliferation of cancer cell lines of different tissue origins was inhibited by the melittin-MIL-2 fusion protein. The melittin-MIL-2 directly inhibited cell proliferation of ovarian cancer cells SKOV3 in a concentration-dependent manner; rIL-2 did not directly inhibit growth of SKOV3 cells in vitro. We observed the maximum inhibitory effect of the fusion protein on cell proliferation after 48 h. The similar inhibitory effects were tested with four additional cancer cell lines of mammary, lung, liver and gastric origins. Our findings indicated that the melittin-MIL-2 also retained its melittin functional activity. In vitro, the melittin-MIL-2 fusion protein may directly kill or mediate immune cells killing the tumor cell lines of different tissue origins.

In the present study, the in vivo data demonstrated that the melittin-MIL-2 had an antitumorigenic effect towards several different human xenograft models and exhibited enhanced antitumor activity compared to rIL-2. IL-2 can motivate immune cells, including T cells, B cells, monocytes, macrophages, LAK cells and NK cells [36, 37]. A mutant IL-2 is ~3000-fold more selective in prompting proliferation of T cells and NK cells in vitro compared with human IL-2 and so is assessed in parallel with IL-2 in mice for antitumor effect. Several mutants recombinant human IL-2 can increase the antitumor effect and minimize systemic toxicity [7–9]. Furthermore, melittin can enhance immune function by increasing Th1 cells function. In addition, several studies have manifested that melittin can induce apoptosis, cell cycle arrest and growth-inhibition in different tumor cells [15, 33–35]. The potent antitumor effect of melittin has been verified in animal models [38–40]. Thus, the melittin-MIL-2 showed significantly enhanced antitumor activity compared to rIL-2. Here, the costimulatory activity of melittin-MIL-2 could cause the additional signal enhancement, suggesting an obvious advantage of the simultaneous presentation of melittin and mutant IL-2 in one molecule.

The tumor microenvironment plays a pivotal role in determining the tumor eradication or dissemination. This environment includes various cell types and soluble components, interacting with each other. In diseased states, a bidirectional network for suppression or progression of tumor tissues is produced by the proportion of immunosuppressive to immunostimulatory cells and components such as cytokines [41–43]. Here, we evaluated the impact of cytokines on tumor microenvironment. The type of cytokines secreted in tumor tissues determines the subsequent type of immune response. Increased concentration of IFN-γ within tumor sites indicates the presence of NK cells in the tumor tissues, which can lead to the expression of MHC molecules on the surface of tumor cells causing increased cell death rooted in higher tumor immunogenicity [44]. Moreover, this case can result in prime CD8^+^ and CD4^+^ cells that are related to efficient tumor inhibition. Along with CD8^+^ and CD4^+^ T lymphocytes, Th0 subtype of T cells promotes the production of INF-γ and suppresses the secretion of IL-4, contributing to the proliferation of Th1 subtype over Th2 cells in the tumor microenvironment. IL-12, one of the potent cytokines, produces a beneficial effect on the secretion of IFN-γ from NK, NKT and Th1 cells in generating antitumor immunity [45–47]. In this study, the significant decline of IL-4 but no change in the production of IL-12 and TNF-α was observed, which showed the activation of Th1 and NK cells in the tumor sites. In short, the melittin-MIL-2 promoted the IFN-γ secretion in tumor tissues and reduced the immunosuppressive cells in vivo. The melittin-MIL-2 treatment could shape a local immunostimulatory microenvironment.

The therapy of localized malignancies has achieved the substantial advances. However, metastatic cancer still requires more effective treatment and remains the primary cause of cancer mortality, including breast cancer. Approximately 90 % of breast cancer-related deaths are attributed to metastasis [48]. It is necessary to effectively improve the prevention or treatment of metastasis and consequently improve the survival of cancer patients. Among cancer immunotherapies, cytokine-based methods have exhibited remarkable anticancer and anti-metastatic effect in numerous experimental studies [49–51]. In particular, IL-2-based immunotherapies have been manifested to produce durable, tumor-specific immune responses capable of preventing recurrence and controlling metastasis. Recent studies have indicated that melittin may induce cytotoxic, antitumor, immunomodulatory, and apoptotic effects in different tumor cells in vivo or in vitro [33, 35, 52]. Furthermore, melittin exhibits the potential of inhibiting cancer cell metastasis [33, 35, 52]. Here, the melittin-MIL-2 inhibited lung metastasis of MDA-MB-231 mammary cancer cells. The recombinant melittin-MIL-2 fusion protein could stimulate tumor-specific immunity and dampen tumor growth and provide systemic protection against metastatic cancer. Therefore, new approaches which prevent or inhibit cancer metastases are of significant clinical value.

## Conclusions

The melittin-MIL-2 fusion protein promoted in vitro expansion and stronger cytolytic activity of activated lymphocytes, especially NK cells. The melittin-MIL-2 induced immune cells killing or directly killed the cancer cell lines of different tissue origins in vitro. The fusion protein showed potent inhibition on the growth of xenograft tumors compared to rIL-2 in vivo. The melittin-MIL-2 treatment could shape a local immunostimulatory microenvironment. Furthermore, the melittin-MIL-2 inhibited lung metastasis of breast cancer. The combination of melittin and a mutant IL-2 should be considered a feasible strategy for enhancing immune cell stimulation and antitumor effects. The melittin-MIL-2 fusion protein is a promising candidate for cancer immunotherapy.
